# Orthodontic traction of impacted canine using magnet: a case report

**DOI:** 10.1186/1757-1626-1-382

**Published:** 2008-12-10

**Authors:** Larry CF Li, Ricky WK Wong, Nigel M King

**Affiliations:** 1 Orthodontics, Faculty of Dentistry, the University of Hong Kong, 2/F, 34 Hospital Road, Sai Ying Pun, Hong Kong SAR, PR China; 2 Paediatric Dentistry, Faculty of Dentistry, the University of Hong Kong, 2/F, 34 Hospital Road, Sai Ying Pun, Hong Kong SAR, PR China

## Abstract

A 15 year and 1 month old Chinese female with palatally impacted upper left canine was successfully treated with an upper removable appliance with a magnet incorporated to provide orthodontic traction force. This case report indicates the possibility of using magnetic force as a safe, effective and comfortable way for orthodontic traction.

## Introduction

The prevalence of palatally impacted canine in the population ranges from 0.27% to 2.4% worldwide [1]. Long and tortuous path of eruption, crowding of the dentition, failure of root resorption of the deciduous canine, trauma and soft tissue pathology have all been considered the reasons for the canine impaction.

If left untreated, an impacted canine could cause morbidity of the deciduous canine, cystic change or crown resorption of itself and/or root resorption of the adjacent lateral incisor [1].

The conventional treatment approach of an impacted canine is to expose the crown of the canine surgically, followed by bonding an attachment onto the crown surface. The attachment is ligated to the arch wire in the mouth, and the tooth can be pulled out with elastics [2]. However, this approach comes with many drawback and limitations such as infection as there is a communication between the attachment and the oral environment gingival inflammation, apical migration of the epithelial attachment, bony recession, exposure of the cementoenamel junction [3], irritation to the lips, difficulty in maintaining oral hygiene and difficulty to adjust and change the direction of force.

Rare earth magnetic alloys have been used in orthodontics increasingly and various authors have reported successful clinical results of the treatment of impacted teeth in humans [3-5]. A magnet, coated with acrylic and attached with a wire extension arm, can be attached to a removable appliance. The position of the magnet can be altered by adjusting the extension arm. By bonding a metal bracket to the impacted tooth after surgical exposure, the impacted tooth will be under magnetic force with a direction controllable by adjustment of the extension arm. This case report describes the use of magnet in the management of an impacted maxillary canine and considers the advantages and limitations of the technique.

## Case presentation

**Figure 1:**
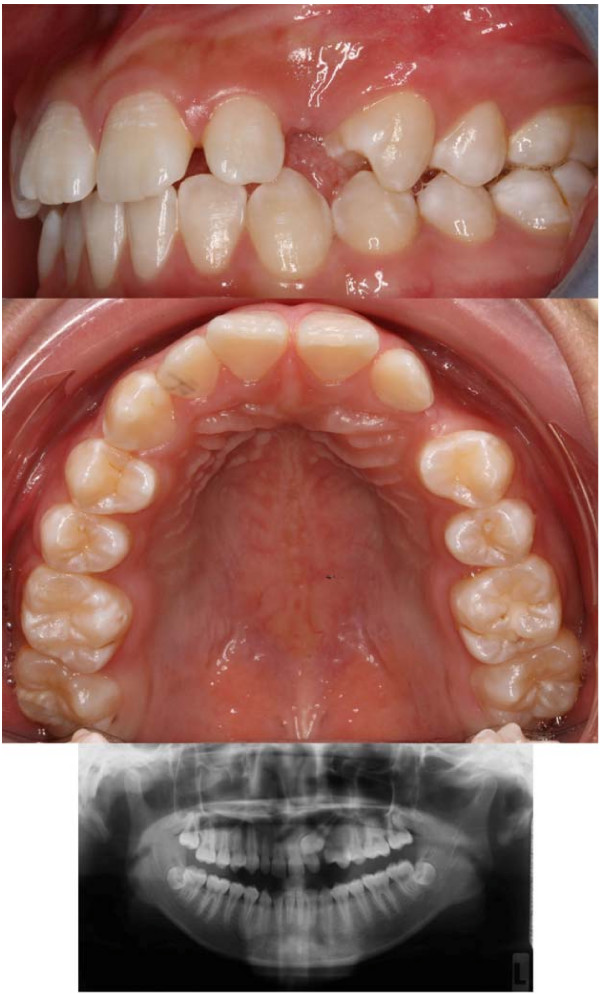
**Pre-treatment intraoral views and panoramic radiograph**. illustrates the intraoral left buccal view (top), upper occlusal view (middle) and panoramic radiograph (bottom) of the patient with an impacted upper left permanent canine. A bulge on the palatal mucosa could be seen palatal to the upper left lateral incisor. The radiograph showed that the impacted canine was mesially inclined and erupting towards the lateral and central incisors.

The patient was a 15 year and 1 month old Chinese female when she presented with a complaint of missing canine. She had an unremarkable medical history. Upon clinical examination, it showed parabolic and symmetrical upper arch, with mild crowding anteriorly. The upper left permanent canine (23) was clinically missing and the adjacent lateral incisor (22) was slightly mobile and mildly inclined distally and labially. A bulge on the palatal mucosa could be palpated behind 22. The radiograph showed impacted 23 which was mesially inclined and was palatal to 22. Half of the root of 22 was resorbed (Figure 1).

**Figure 2:**
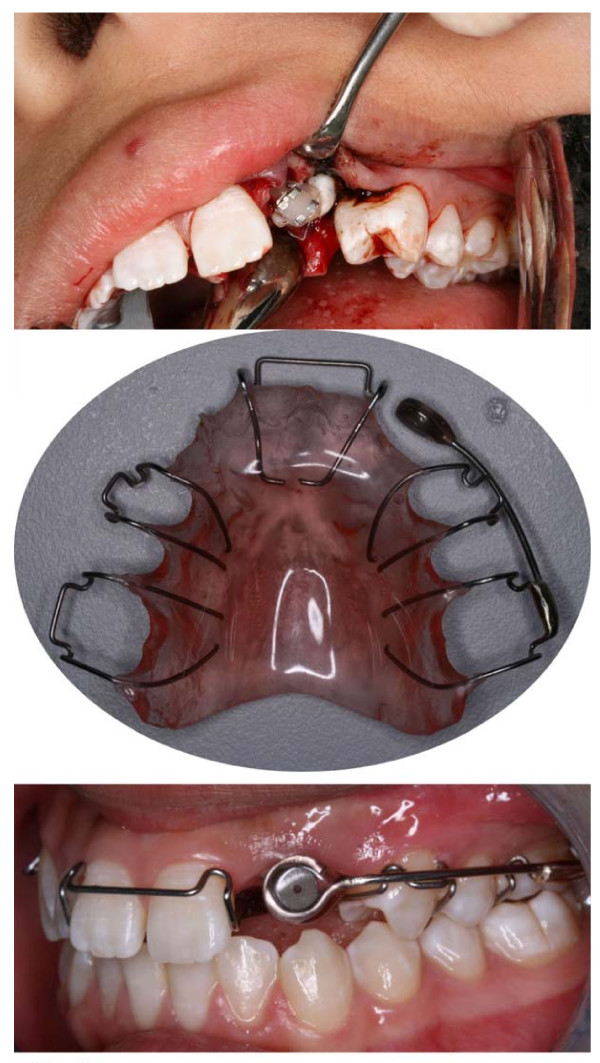
**Surgical exposure and orthodontic traction with a magnetic removable appliance**. illustrates the surgical exposure and bonding of a metal bracket to the impacted canine (top), the removable appliance with a magnet (middle) and the magnet attached to an adjustable extension arm on the appliance (bottom).

The treatment plan was extraction of 22 and surgical exposure of 23 followed by orthodontic traction with magnet. During the surgical exposure, a metal bracket was bonded to the crown of 23 and the wound was closed with suture. 2 weeks later, a specially designed upper removable appliance with a magnet arm where the magnet (MagneForce, ORMCO, USA) was acrylic coated to prevent direct contact with the oral environment, extending to the impacted canine area was issued to the patient. The magnet was carefully orientated to ensure the flat attractive surface was as near parallel as possible to the metal bracket bonded to the tooth. The patient was instructed to wear the appliance on a full day basis (Figure 2).

**Figure 3:**
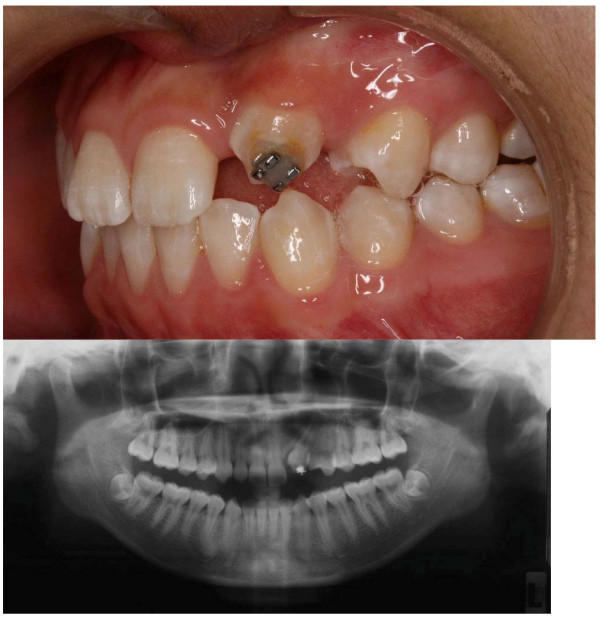
**The post-treatment view and radiograph**. illustrates the left buccal intraoral view (top) and panoramic radiograph (bottom) of the patient after magnetic traction of the impacted upper left permanent canine. Around two-third of the crown of the upper left canine had erupted. The radiograph showed that the canine was erupting towards a more distal direction.

Three months after wearing the appliance, 3 mm of 23 had erupted above the gingivae. The magnet arm was then adjusted to guide the tooth to erupt more distally and buccally. At the 12^th ^month, two third of the crown had erupted and the patient was ready to receive simple fixed appliance orthodontic therapy to further align the tooth into the arch (Figure 3). No adverse effect was observed.

## Discussion

Magnetic forces from rare earth magnets are generated along the line of a magnetic plane, and therefore, it is possible to prescribe tooth movement in all three planes of space by altering the magnet arm [6]. Moreover, the attractive force could effect through the mucosa thus simulating a normal eruption process. An attractive force level of 0.2 to 0.5 N was proved to be effective [6].

In the case described above, orthodontic traction of the impacted canine using a magnetic force minimized the risk of gingival recession by allowing the tooth to erupt through the attached gingivae and also reduced the time for the patient undergoing a fixed appliance treatment. The patient was able to maintain a good oral hygiene and she showed high compliance with this removable appliance.

However, magnetic traction still carries limitations with its use. Magnetic attractive forces are inversely proportional to the square of the distance. This means the magnets have to be placed proximal enough to each other, otherwise there will be dramatic drop in force level [7]. In addition, the magnets corrode significantly in the intra-oral environment, and have to be coated in acrylic carefully, which may increase its bulkiness and lead to patient's discomfort.

## Conclusion

This case reports suggests that magnetic traction with an removable appliance can be a safe, effective and comfortable method to disimpact a maxillary canine and merits further investigation.

## Consent

Written informed consent was obtained from the patient for publication of this case report and accompanying images. A copy of the written consent is available for review by the Editor-in-Chief of this journal.

## Competing interests

The authors declare that they have no competing interests.

## Authors' contributions

RWKW designed the removable appliance with magnetic arm and supervised the treatment to the patient. LCFL conducted the treatment and was a major contributor in writing the manuscript. NMK was responsible for the surgical part of the procedure. All authors read and approved the final manuscript.

## Authors' information

Larry C.F. Li BDS (HK) Ricky W.K. Wong* BDS (HK), MOrth (HK), PhD (HK), FRACDS, MOrthRCS (Edin), FHKAM (Dental Surgery), FCDSHK (Orthodontics)Nigel M King BDS(Lond), MSc (Lond), PhD (HK), LDS RCS (Eng), Hon FDSRCS (Edin), FHKAM (Dental Surgery), FCDSHK (Paediatric Dentistry) LCFL is a postgraduate student in Master of Orthodontics, Faculty of Dentistry, the University of Hong. RWKW is an associate professor in Orthodontics, Faculty of Dentistry, the University of Hong Kong. NMK is a professor in Paediatric Dentistry, Faculty of Dentistry, the University of Hong Kong.
